# Immunological blocking of spermidine‐mediated host–pathogen communication provides effective control against *Pseudomonas aeruginosa* infection

**DOI:** 10.1111/1751-7915.13279

**Published:** 2018-05-14

**Authors:** Jianhe Wang, Jing Wang, Lian‐Hui Zhang

**Affiliations:** ^1^ Guangdong Province Key Laboratory of Microbial Signals and Disease Control State Key Laboratory for Conservation and Utilization of Subtropical Agro‐Bioresources College of Agriculture South China Agricultural University Guangzhou 510642 China; ^2^ Institute of Molecular and Cell Biology 61 Biopolis Drive Singapore 138673 Singapore

## Abstract

*Pseudomonas aeruginosa* is known to cause life‐threatening infections. The previous studies showed that the type III secretion system (T3SS) of this pathogen is a key virulence determinant, which is activated by polyamines signals spermidine (Spd) and spermine (Spm) from mammalian host. To test the potential of blocking host–pathogen communication in disease control, in this study we developed a high potency mouse monoclonal antibody (Mab 4E4, IgG1 sub‐isotype) by using Spm–protein conjugate as an immunogen. Antibody specificity analysis showed that the antibody specifically recognize Spd and Spm. *In vitro* study showed the antibody significantly protected A549 cells against *P. aeruginosa* infection, and this protection was achieved by blocking polyamine uptake and downregulating T3SS expression. *In vivo* single injection of mouse with Mab 4E4 drastically reduced the serum polyamine level, which was maintained for more than 1 week. In a murine model of *P. aeruginosa* acute infection, injection of Mab 4E4 protected mice from lung injury and significantly improved the survival rate of mice.

## Graphical abstract

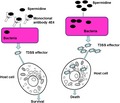

## Introduction

*Pseudomonas aeruginosa* is an important opportunistic human bacterial pathogen that can cause severe infections in cystic fibrosis patients and immunocompromised individuals (Bodey *et al*., 1983; Richards *et al*., 2000), which has a high mortality rate despite aggressive treatments with antimicrobial drugs (Craven and Steger, 1996; Wunderink, 1995). One of the abilities of this bacterium to cause epithelial injury and to avoid host innate immune responses is majorly due to expression of the toxic effector proteins that are translocated directly into eukaryotic cells via a type III secretion system (T3SS) (Kudoh *et al*., 1994; Yahr *et al*., 1997; Finck‐Barbancon *et al*., 1997; Sawa *et al*., 1998, 1999). Numerous studies have demonstrated that T3SS is a key virulence determinant of *P. aeruginosa* that plays a critical role in establishing acute infections by manipulating eukaryotic host cell responses (Engel and Balachandran, 2009; Hauser, 2009).

T3SS, which is widely conserved in a broad range of pathogens, is a specialized needle‐like structure that delivers toxic effector proteins directly from pathogen into host cells in a highly regulated manner. Over 40 T3SS genes have been reported in *P. aeruginosa,* and the transcriptional expression of these T3SS genes are controlled by the master regulator ExsA, which activates the T3SS by interacting with the promoters of known T3SS genes (Hauser, 2009; Diaz *et al*., 2011). The expression and activity of ExsA encoded by the *exsCEBA* operon are modulated by a range of regulators, including ExsC, ExsD, ExsE, Vfr, RtsM, RetS and GacA (Diaz *et al*., 2011), suggesting that the T3SS is tightly controlled by stringent and complex regulatory mechanisms.

### New

Our previous study showed that spermidine, which is produced by both eukaryotic and prokaryotic organisms, is the key mammalian host signal that induces T3SS gene expression (Zhou *et al*., 2007). Deletion of the spermidine transporter encoded by *spuDEFGH* significantly decreased T3SS gene expression and attenuated the cytotoxicity of *P. aeruginosa* on Hela cell lines.

Bioinformatic analysis suggests that SpuDEFGH might form an ABC transporter system for spermidine uptake, in which SpuD and SpuE are the periplasmic spermidine‐preferential binding proteins, SpuF is the ATPase, and SpuG and SpuH form the transmembrane channel (Lu *et al*., 2002; Zhou *et al*., 2007). The subsequent crystal structural analysis showed that SpuE is a spermidine‐specific substrate‐binding protein (Wu *et al*., 2012). Significantly, null mutation of SpuE decreased the host cell extract‐dependent induction of T3SS system and attenuated the bacterial cytotoxicity (Zhou *et al*., 2007), indicating that the pathogen could sense and cunningly exploit the spermidine molecules produced by mammalian host to induce T3SS gene expression. Given the importance of T3SS in *P. aeruginosa* pathogenesis, over the past few years, over a dozen of chemical inhibitors and two types of antibodies have been evaluated *in vitro* for their capacity to inhibit T3SS gene expression, and to protect host cells from the T3SS‐mediated cytotoxicity (Anantharajah *et al*., 2016). These inhibitors and antibodies act either by interference of T3SS regulators or by blocking T3SS translocon apparatus, but only a few have been tested under *in vivo* conditions. To test whether interference of spermidine‐dependent host–pathogen signalling could attenuate *P. aeruginosa* virulence, we recently designed and synthesized a range of spermidine derivatives, with an aim to block spermidine transportation, and found one inhibitor displaying potent anti‐infection activities (Wang *et al*., 2016). The results highlight the promising potential of developing anti‐infection therapies by abrogation of the cell–cell signalling system that modulates bacterial T3SS expression. In this study, we report development of a mouse monoclonal antibody (Mab 4E4, IgG1 sub‐isotype) against spermidine (Spd). The antibody specificity was evaluated by using a range of Spd analogues. *In vitro* study showed that addition of Mab 4E4 significantly reduced the cytotoxicity of *P. aeruginosa* on A549 cells by blocking Spd uptake and downregulating T3SS expression. A single injection of Mab 4E4 could substantially reduce the serum Spd level of mouse for over 1 week, and significantly improved the survival of mice against *P. aeruginosa* infection.

## Results

### Production and characterization of Spd‐specific Mabs

BALB/c mice were immunized with HSA–Spd conjugate (Fig. S1), and their spleens were removed for fusion to myeloma cells. OVA–Spd conjugate was used as coating antigen for ELISA screening, which led to identify two positive hybridomas cell lines (Mab 4E4 and Mab 3H4) reacting to both antigens. The cell culture supernatants of Mab 4E4 and Mab 3H4 were titrated against Spd to determine the relative affinity. The results indicate that both Mab 3H4 and Mab 4E4 are highly potent Mabs with affinity at 10^9^ m^−1^ and 10^11^ m^−1^ respectively.

**Figure 1:**
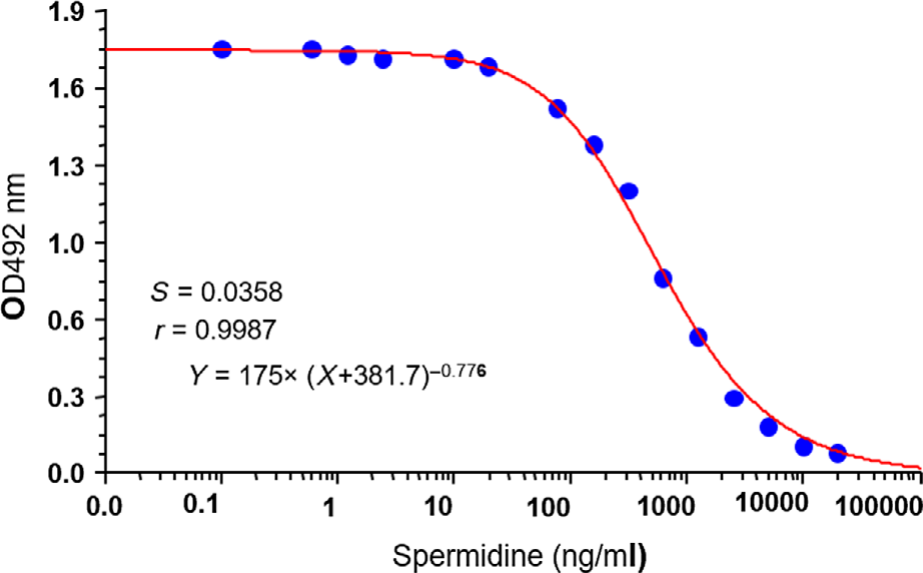
The calibration curve of spermidine for Mab 4E4. The experiment was repeated for at least three times and one set of representative data was shown here.

For further characterization, Mab 4E4 and Mab 3H4 clones were cultured. The supernatants were collected, and the immunoglobulin was purified using ammonium sulfate precipitation and the protein A coupled agarose column. The purity was verified using SDS‐PAGE gel electrophoresis (Fig. S2). The class and subclass of Mabs were then determined and the results showed that both Mabs are of IgG1 subclass and contain kappa light chain (Fig. S3; Table 1). The inhibition tests of spermidine and the other four polyamine compounds, including ornithine, putrescine, cadaverine and spermine, showed that only spermine and spermidine could specifically inhibit the binding of Mab 4E4 to the coated OVA–Spd (Table 2), indicating that only Spm and Spd contain the structural motif essential for Mab 4E4 recognition (Fig. S4). Determination of calibration curve of Spd found that Mab 4E4 could detect Spd as low as 20 ng ml^−1^ (Fig. 1).

**Table 1:** Competitive ELISA analysis of molecular interaction of Mab 4E4 with Spd analogues.^a^

Chemical	Molecular formula	Mab 4E4	
		ID50^a^ (nmol)	Cross‐reaction (%)
Ornithine	C_5_H_12_N_2_O_2_	34.1	0.0016
Putrescine	NH_2_(CH_2_)_4_NH_2_	28.2	0.0020
Cadaverine	NH_2_(CH_2_)_5_NH_2_	21	0.0026
Spermine	NH_2_(CH_2_)_3_NH(CH_2_)_4_NH(CH_2_)_3_NH_2_	0.54 × 10^−3^	100
Spermidine	NH_2_(CH_2_)_3_NH(CH_2_)_4_NH_2_	0.53 × 10^−3^	98

a ID50 means 50% inhibitory dose in the competitive ELISA assay. The experiment was repeated three times with similar results. The table lists a set of representative data.

**Table 2:** Identification and characterization of monoclonal antibodies.^a^

Hybridoma	Class and subclass	Type	Titre of cell culture supernatant	Affinity constant (M^−1^)
Mab 4E4	IgG1	kappa	1:40,000	7.3 × 10^11^
Mab 3H4	IgG1	kappa	1:80,000	5.1 × 10^9^

a The experiment was repeated three times and the table lists a set of representative data.

### Western blot analysis of Mab 4E4 detection of Spd and Spm

**Figure 2:**
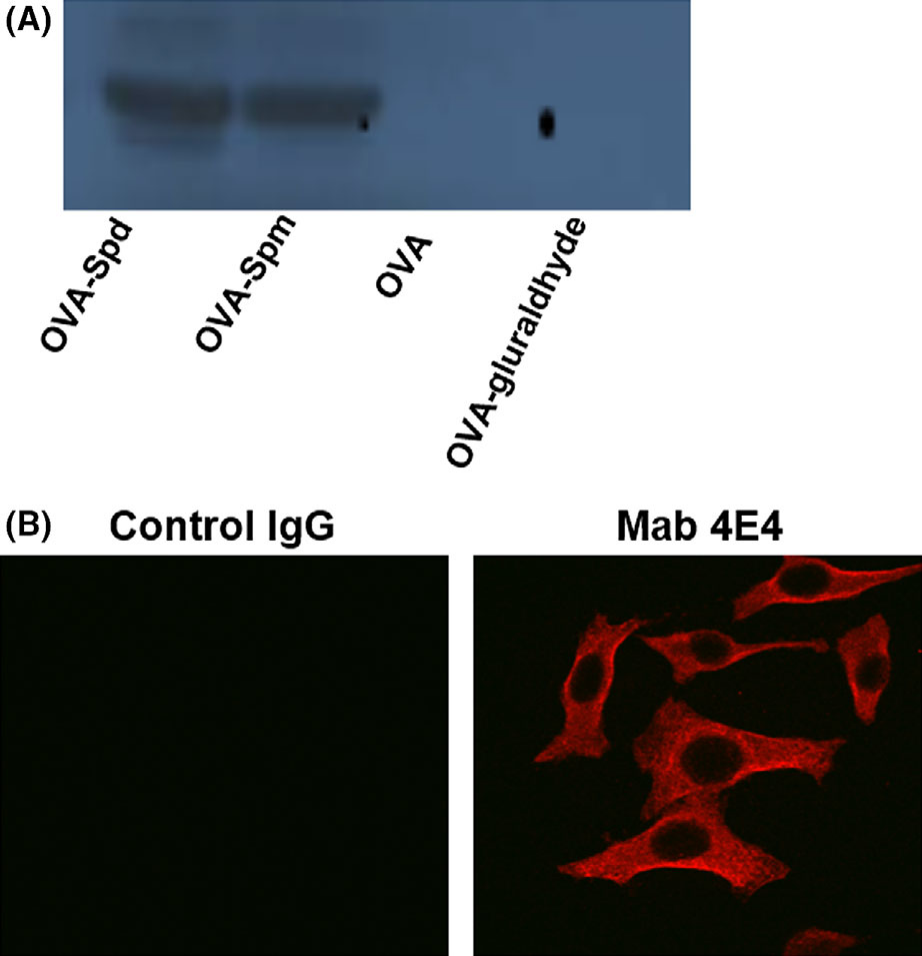
Characterization of monoclonal antibody Mab 4E4. (A) Mab 4E4 recognized Spd and Spm, but not OVA. (B) Spermidine in A549. Mab 4E4 was used in 1:2000 ratio.

To further confirm the ability of Mab 4E4 in recognition of Spd and Spm, samples containing same amount of OVA–Spd, OVA–Spm, OVA and OVA–glutaraldhyde were subjected to SDS‐PAGE electrophoresis and Western blot analysis using Mab 4E4. The results showed that Mab 4E4 could detect protein samples containing Spd and Spm, but not OVA and OVA–glutaraldhyde (Fig. 2A). Previous study indicated that Spd and Spm are normally present at millimolar concentrations in plants and animals (Igarashi and Kashiwagi, 2000). We then tested whether Mab 4E4 could detect these polyamine molecules in mammalian cell lines. *In situ* Western blot results showed that Spd and Spm signals were evenly distributed in the cytosol of human cell line A549, whereas control IgG could not detect these polyamine molecules (Fig. 2B).

### Mab 4E4 blocks the transcriptional expression of T3SS genes

**Figure 3:**
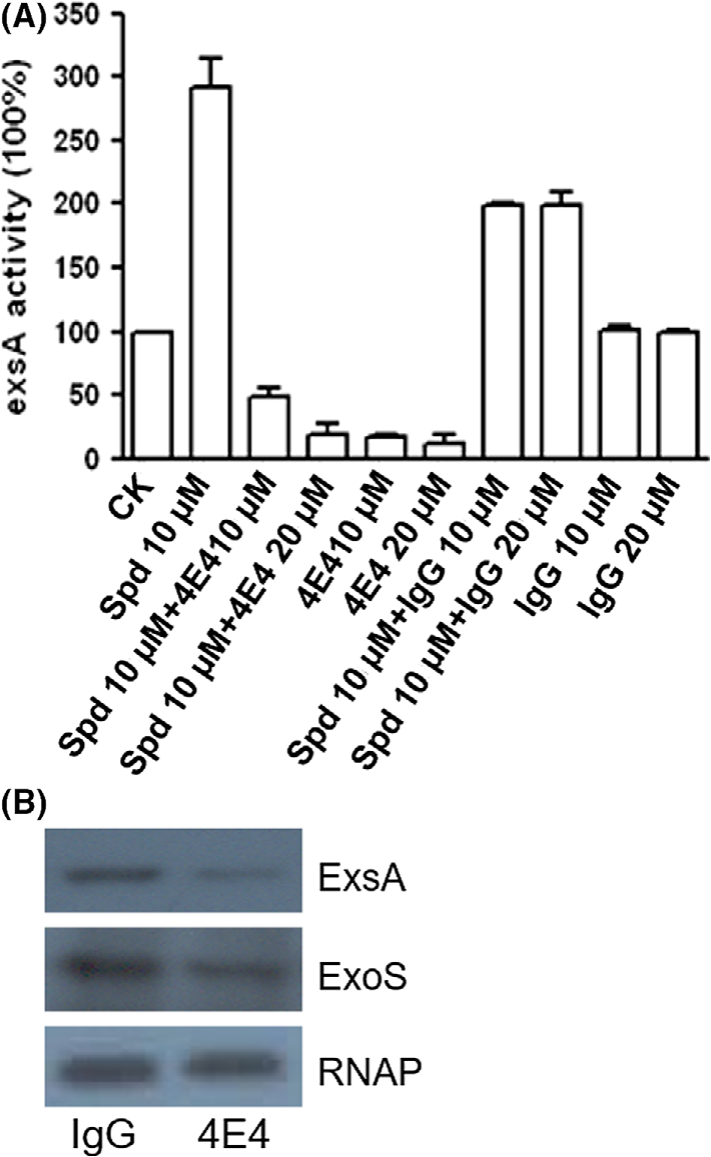
Effect of Mab 4E4 on T3SS gene expression in *Pseudomonas aeruginosa*. (A) Exogenous addition of Mab 4E4 suppressed the Spd (10 μM)‐induced T3SS expression. The final concentrations of Spd and Mab 4E4 were 10 and 20 μM respectively. The data were the means of three replicates. (B) Immunoblotting detection of T3SS global regulator ExsA and the effector ExoS. Bacterial cultures were grown in minis medium supplemented with NTA and 10 μM Spd, then 20 μM Mab 4E4 and IgG were added respectively. The protein samples from bacteria were separated by 10% SDS‐PAGE electrophoresis. The experiments were repeated for at least three times with similar results.

To determine the effect of Mab 4E4 on suppression of T3SS gene expression, the T3SS reporter strain PAO1pClacZ (Table S1), which was generated by placing the *lacZ* gene under the control of the *exsCEBA* promoter (Zhou *et al*., 2007), was grown in MINIS medium supplemented with spermidine in the presence or absence of Mab 4E4 by using non‐specific IgG as a control. After growth for 4 h, the bacterial cells were collected by centrifugation and the β‐galactosidase enzyme activity encoded by *lacZ* under the control of *exsCEBA* promoter of T3SS was determined. As reported previously (Zhou *et al*., 2007), addition of Spd at a final concentration of 10 μM substantially induced the expression of *exsA* compared with the control without Spd (CK) (Fig. 3A). Addition of Mab 4E4 suppressed the Spd‐induced expression of *exsA* in a dosage‐dependent manner (Fig. 3A). In contrast, addition of non‐specific IgG partially reduced the level of exsA expression, likely due to non‐specific binding of polyamine to proteins (Tabor and Tabor, 1984; Schuber, 1989), but was not able to reverse completely the Spd‐induced expression of *exsA* (Fig. 3A). The results were further confirmed by immunoblotting analysis, which showed that the protein levels of the T3SS regulator ExsA and effector ExoS were substantially reduced by co‐culture of the bacterial pathogen with 20 μM Mab 4E4 compared with IgG control (Fig. 3B).

### Mab 4E4 attenuates the cytotoxicity of *P. aeruginosa* on host cells

**Figure 4:**
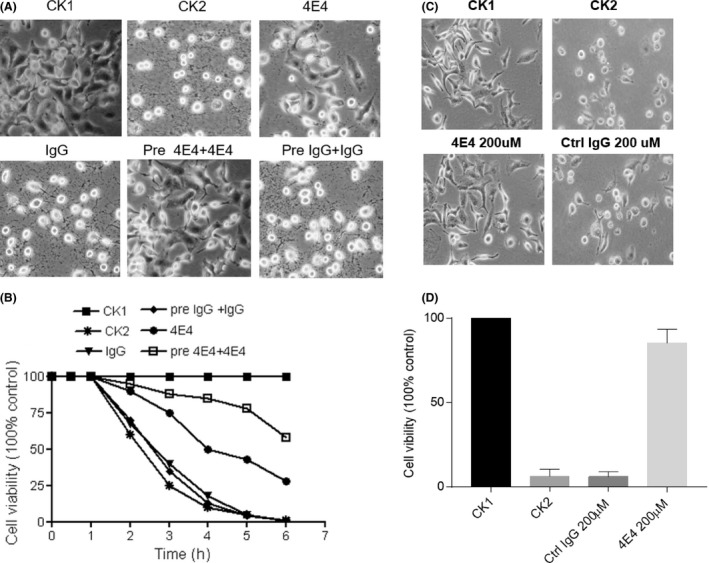
Mab 4E4 attenuates the cytotoxicity of *Pseudomonas aeruginosa* on mammalian cell line A549. (A) Photograph images of A549 cells without infection (CK1); A549 infected by *P. aeruginosa* PAO1 (CK2); A549 Cells inoculated with PAO1 plus 200 μM Mab 4E4 (4E4) or IgG (IgG); A549 cells infected by PAO1 pre‐treated 2 h with Mab 4E4 (Pre 4E4 + 4E4) or IgG (Pre IgG+IgG). PAO1 MOI = 50, photos were taken under microscope (10 × objective). (B) Effect of 4E4 on cell viability in A549 cell against PAO1 infection. Viability was measured by WST‐1 test. Data are expressed as percentage on internal control for each cell type measured after over 12 h after cell seeding (time 0) and are the mean ± SEM of three replicates. The experiments were repeated for three times. (C) Effect of Mab 4E4 on cell viability in A549 cell against PA14 infection. Cells were treated with Mab 4E4 or control IgG (4E4 200 μM or control IgG 200 μM) in association with PA14 (MOI = 50) for 3 h compared with normal cells without infection and IgG treatment (CK1) and only infected PA14 without IgG treatment (CK2). Photos were taken under microscope (10 × objective). (D) Effect of 4E4 on cell viability in A549 cell against PA14 infection. Cells were treated with different dosages of 4E4 or control IgG in association with PA14 (MOI = 50) compared with normal cell or PA14 infection alone cell. Viability was measured by WST‐1 test. Data are expressed as percentage on internal control for each cell type measured after over 12 h after cell seeding (time 0) and are the mean ± SEM of three replicates. The experiments were repeated for three times.

As Mab 4E4 could reverse exogenous Spd activity and inhibit T3SS expression, we set out to determine whether exogenous addition of Mab 4E4 could reduce the bacterial cytotoxicity and protect host cells against *P. aeruginosa* infection. The bacterial strain PAO1 or PA14 was cultured in LB medium supplemented with the chelating reagent nitrilotiracetic acid (NTA) at a final concentration of 7.5 mM, which is the condition inducing T3SS expression (Zhou *et al*., 2007). When Mab 4E4 was added to bacterial solution at the time of inoculation, the treatment increased the cell viability by 35% compared to the control treated with IgG, whereas pre‐treatment of the pathogen with Mab 4E4 for 2 h before infection increased the viability of host cells up to 85% (Fig. 4).

### Mab 4E4 injection decreases mouse serum Spd level and protects animals against bacterial infection

**Figure 5:**
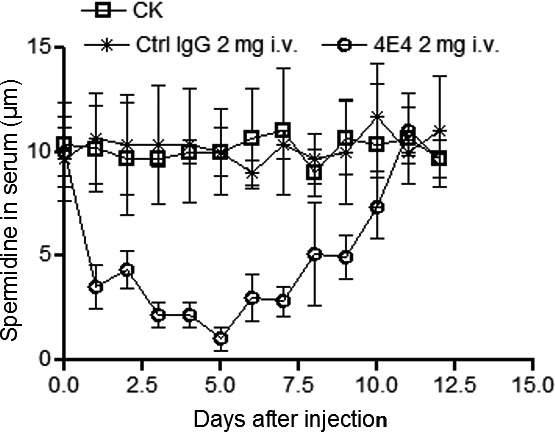
Spermidine levels in mouse serum after injection with Mab 4E4 and IgG respectively. Ctrl IgG and Mab 4E4 (2 mg per mouse, *n* = 10) were introduced into mouse through I.V. injection. The data are the means of three replicates and the experiments were repeated three times.

**Figure 6:**
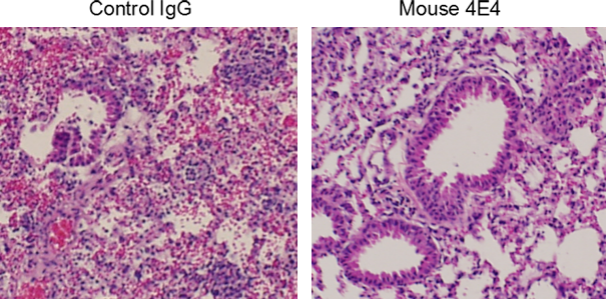
Mab4E4 protects mouse lung damages caused by *P. aeruginosa* infection. The mice were treated with Mab 4E4 and control IgG (i.v injection, 2 mg per mouse) 2 h prior to *P. aeruginosa* infection. Lung sections were haematoxylin–eosin stained for 24 h after infection with *P. aeruginosa* PAO1. Magnification of objective lens 20×.

To determine the effect of Mab 4E4 on mouse Spd level under *in vivo* conditions, the control IgG and Mab 4E4 were injected through the tail vein of mice. The Spd concentration in mouse serum was quantified at different time points post injection (Fig. 5). Significantly, Mab 4E4 caused a substantial decrease of serum Spd level for at least 1 week, whereas IgG treatment did not obviously alter the concentration of Spd in the animal serum. We then compared the lung histology between the mice treated with Mab 4E4 and the mice treated with control IgG (Fig. 6). The mice injected with control IgG 2 h after bacterial instillation showed severe neutrophil recruitment and destruction of alveolar structures; the mice treated with Mab 4E4 had almost no neutrophils in their airspaces and maintained normal alveolar structures.

**Figure 7:**
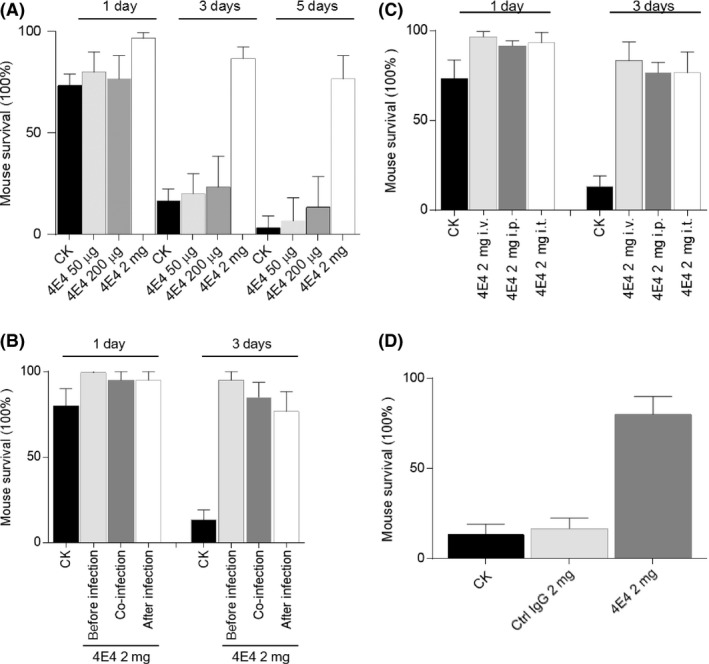
The effect of Mab 4E4 on survival of Balb/c mice against PAO1 infection. (A) The effect of different dosages of Mab 4E4 on mice survival rate. Three dosages of Mab 4E4 (50 μg, 200 μg and 2 mg) were injected into mice (*n* = 10 per group) by i.v., respectively, at 2 h before bacterial infection (1.5e6 CFU). (B) The effect of different time points of Mab 4E4 (2 mg per mouse) injection on mice (*n* = 10 per group) survival rate. Mice were injected with Mab 4E4 2 h before, 2 h later or at the same time with PAO1 challenge (1.5e6 CFU). (C) The effect of Mab 4E4 delivery ways (i.v.; i.t.; i.p.) of Mab 4E4 on mice (*n* = 10 per group) survival rate. (D) The effect of Mab 4E4 on mouse survival against PAO1 infection compared with control IgG. Data are average ± SD and were analysed by using Prism7 GraphPadby. Data are representative of three independent experiments.

Mab 4E4 was tested for its ability to protect mice against *P. aeruginosa* infection. The suitable concentration of Mab 4E4 in protection of animal was first investigated. The results showed that Mab 4E4 dosage below 200 μg had no or minor protective effect *in vivo*, while injection of 2 mg Mab 4E4 per animal increased the animal survival rate by about 80% compared with the PBS buffer control (CK)_(Fig. 7A) or the IgG control (Fig. 7D).

In another set of experiments, Mab 4E4 was administered by an intravenous injection 2 h before, after or at the same time of bacterial lung instillation (fourfold lethal dose). The results showed that Mab 4E4 injection 2 h before bacterial inoculation showed better protective efficiency than the other two treatments (Fig. 7B). The efficacy of intraperitoneal (i.p.), intravenous (i.t.) and Intrathecal delivery of Mab 4E4 also were compared. Intravenous (i.v.) injection of Mab 4E4 delivered a slightly higher protective efficiency than the other two against PAO1 infection (Fig. 7C).

## Discussion

*Pseudomonas aeruginosa* is one of the main pathogens responsible for nosocomial infections due to its ability to survive and multiply in diverse environments and its notorious resistance to various common antibiotics. In this study, we report the development of the Spd‐specific Mab 4E4, with an aim to block Spd‐mediated induction of *P. aeruginosa* T3SS expression, which is the key virulence determinant of the bacterial acute infection (Hauser, 2009; Galle *et al*., 2012). The potent monoclonal antibody Mab 4E4 displayed a strong affinity to Spd and Spm, which are widely presented in plants and mammalian organisms (Larqué *et al*., 2007; Lu, 2002), and shown to be the potent host signals capable of inducing transcriptional expression of T3SS genes in *P. aeruginosa* (Zhou *et al*., 2007; Wu *et al*., 2012). Our results showed that introduction of Mab 4E4 into mice could substantially reduce the free Spd level in the animal serum and significantly increase the animal survival rate against *P. aeruginosa* infection.

The findings from this study provide further evidence to support the key role of Spd‐mediated host–pathogen communication in modulation of T3SS gene expression in *P. aeruginosa*. The current knowledge indicates that the T3SS of *P. aeruginosa* are modulated by complicated regulatory mechanisms. A range of intrinsic and extrinsic regulators or environmental cues, including Spd and Spm, are known to affect the expression or function of the bacterial T3SS (Zhou *et al*., 2007; Diaz *et al*., 2011). The previous studies indicate that the host Spd signals are transported through a high affinity ABC transporter encoded by the *spuDEFGH* gene cluster (Zhou *et al*., 2007; Wu *et al*., 2012). Intriguingly, the *speD* and *speE* genes of *P. aeruginosa* are known to encode Spd biosynthesis (Lu *et al*., 2002), but deletion of these two genes did not seem to affect T3SS expression under *in vitro* culture conditions (Zhou *et al*., 2007). The likely reason is that the yield of Spd production *in vitro* is too low to exert any effect on T3SS expression. The findings from this study seem to indicate unequivocally that the same applies under *in vivo* conditions, and the endogenous Spd molecule produced by host organism is the key source of signal that induces the T3SS expression in *P. aeruginosa*.

Given that Spd signal uptake relies on the SpuDEGFH ABC transporter, in which the substrate‐binding protein SpuE interacts with Spd through an “open‐to‐closed” conformational switch with the resultant closed ligand‐bound form (Wu *et al*., 2012), it was thought that certain Spd structural analogues may be able to stuck to SpuE and thus blocking Spd signal transportation (Wang *et al*., 2016). The rational design led to identify an inhibitor R101SPM, a conjugate of Spm and a bulky molecule rhodamine, which showed a potent inhibitory activity on *P. aeruginosa* T3SS expression and the bacterial virulence (Wang *et al*., 2016). While the detailed mechanism of inhibition remains to be further characterized, the available evidence suggests that R101SPM acts by blocking signal transportation. In this study, Mab 4E4 was generated by using HAS–Spd as an antigen, which displays an ultra‐high affinity (7.3 × 10^11^ m^−1^) to Spd. The findings that Mab 4E4 could effectively quench Spd signals thus present another feasible and promising approach to control *P. aeruginosa* infections by interference of the Spd‐mediated host–pathogen chemical communications.

In summary, we report the generation and characterization of an ultra‐high affinity monoclonal antibody Mab 4E4 specific for Spd and Spm, and present evidence that Mab 4E4 could suppress Spd‐mediated T3SS expression and have promising therapeutic potentials for control and prevention of *P. aeruginosa* infections. The fact that a single injection of Mab 4E4 could effectively quench and maintain serum Spd at a low level for about 1 week may offer a valuable window of time for host innate immune systems to get rid of invaders and recover from bacterial infections. In addition, Mab 4E4 can also be used as a useful immunological agent to establish sensitive, specific and reproducible immunoassay methods for investigation and characterization of Spd and Spm, whose biological functions and associated mechanisms in living organisms remain largely undetermined. Since Spd has many other important functions in the host, free Spd needs to be maintained at an appropriate level for the host necessitating a careful dosing of Mab4E4 in further in vivo studies. Should this approach qualify for human application, the development of a humanized Mab4E4 derivative will become necessary.

## Experimental procedures

### Animals for antibody preparation

BALB/c mice were housed under pathogen‐free conditions with unlimited access to food and water (BRC, A‐STAR, Singapore). All mice used for this study were 5–6 weeks old with body weights ranging from 22 to 25 g. All animal protocols were approved by the Institutional Animal Care and Use Committee (IACUC) of the Agency for Science, Technology and Research, Singapore.

### Preparation of HSA–Spd and OVA–Spd

For raising antibody, Spd needs to be attached to a carrier protein. The following method was used for preparation of Spd conjugates. Human serum albumin (HSA) and ovalbumin (OVA) (10 mg ml^−1^ in 0.1 M carbonate buffer, pH 9; NaCl 0.9%; sodium dodecyl sulfate [SDS], 0.1%) was incubated separately with glutaraldehyde (20 μl of 25% glutaraldehyde aqueous solution) for 1 h at room temperature in the dark. The mixtures were then separated on Sephadex G‐25 column conditioned with carbonate buffer to eliminate the glutaraldehyde excess. The peak corresponding to activated HSA or OVA was incubated with an excess amount of Spd in carbonate buffer for 16 h at room temperature in the dark. Finally, the reaction was blocked by addition of glycine (1 M, final concentration) for 6 h, and the solution was extensively dialysed against phosphate‐buffered saline (PBS). The conjugates were lyophilized to dryness and stored at −20°C till further use.

### Production of monoclonal antibodies (Mabs)

BALB/c mice were subcutaneously immunized with 200 μg of HSA–Spd conjugates in Freund's complete adjuvant. Four weeks later, the mice were consecutively given three times booster immunizations with the half dose of antigen emulsified in Freund's incomplete adjuvant at 2‐week interval. Spleens were removed from immune animals 3 days after the last time booster injection. A single cell suspension was prepared from each spleen in 5 ml of tissue culture medium (Dulbecco's MEM containing 4.5 g L^−1^ glucose, 10% fetal calf serum [FCS]). Tissue culture medium constituents and FCS were purchased from Gibco. Red blood cells were removed from the spleen cell suspension in the lysis step. The cells were underlaid with 10 ml of FCS prior to centrifugation at 800 g. The supernatant was discarded, and the cell pellet was suspended in 10 ml of medium. Cell fusion with myeloma cells (SP2/0) was performed by a conventional technique to generate hybridoma cells. Supernatants of the cells were tested using ELISA. Selected clones were subcloned by limiting dilution.

### Mabs detection and characterization

The checker board procedure was used to optimize the coating antigen concentrations. To screen antibody in the supernatant of cell culture medium, OVA–Spd were coated onto the wells of microtitre plates. Supernatants of the wells containing a monoclonal cell growth were characterized for titre, specificity and affinity (Beatty *et al*., 1987). Selected antibody‐producing clones were cultured in 150 ml flasks. Supernatants were collected and purified using ammonium sulfate precipitation and the protein A coupled agarose column. Purified immunoglobulin was used to establish a calibration curve in culture supernatant. For cross‐reactivity study, supernatants of cell culture medium with proper dilutions were reacted with Spd or other polyamine compounds (100 pg ml^−1^ to 10 μg ml^−1^) and transferred into the OVA–Spd conjugate precoated microtitre wells, washed and added to HRP‐IgG, and incubated for 30 min. The colorimetric reaction was stopped by adding 50 μl of 2 M H_2_SO_4_ solution, and the ELISA assay was conducted following the standard protocol. Mabs class and subclass determination was carried out using Pierce ImmunoPure monoclonal antibody isotyping kit (HRP/ABTS) as instructed by the manufacturer. The commercial mouse IgG (Santa Cruz Biotechnology) was used as a control for specificity analysis.

### Cytotoxicity assay

To determine the cytotoxicity of *P. aeruginosa* strain PAO1 or PA14, A549 cells were seeded in 96‐well tissue culture plates containing 100 μl of Dulbecco's modified Eagle medium (DMEM) and allowed to grow at 37°C for 16 to 18 h to obtain 80–90% monolayer confluency (about 1.0 × 10^4^ cells/well). Culture supernatants were removed, the monolayer was washed once with PBS buffer. For inoculation, the fresh bacterial cells were re‐suspended and diluted in DMEM medium to a concentration about 1 × 10^7^ CFU per ml or otherwise indicated. Thereafter, 100 μl of the bacterial dilution was either incubated with Mab 4E4 at a final concentration of 200 μM for 2 h prior to inoculation, or applied at the same time with Mab 4E4 to the A549 cell monolayers at a multiplicity of infection (moi) of 50. In this experiment, same amout of IgG was used as a non‐specific control. After infection for 4 h at 37°C, A549 cell viability was determined by WST‐1 assay, which quantifies mitochondrial metabolic activity, following the manufacturer's instructions (Roche).

### Quantitative β‐galactosidase assay

The T3SS reporter strain PAO1 pClacZ (Zhou *et al*., 2007) was grown overnight in LB liquid medium supplemented with 7.5 mM NTA. The bacterial cells were diluted 1:200 to fresh MINIS medium supplemented with spermidine at a final concentration of 10 μM, IgG, Mab 4E4, IgG +spermidine or Mab 4E4 + spermidine respectively. The growth was continued with shaking at 37°C for 4 h to allow OD_600_ reaching about 1.0. The β‐galactosidase activity was measured as previously described (Sambrook *et al*., 1987). The experiment was repeated for at least three times and the data shown are the means of three replicates and given as percentage of *exsA* expression by setting blank control as 100%.

### Protein isolation and Western blotting analysis

For Western blot analysis of Mab 4E4 interaction with Spd and Spm, samples containing 10 μg of OVA–Spd, OVA–Spm, OVA, OVA–glutaraldhyde were loaded respectively for SDS‐PAGE electrophoresis analysis. Western blot analysis was performed following the standard protocol by using purified Mab 4E4. For western blotting analysis of the T3SS expression, overnight bacterial cultures were inoculated at a 1:200 ratio to fresh LB medium supplemented with NTA, Mab 4E4 or control IgG as indicated. After incubation at 37°C for 4 h, the bacterial cultures were chilled on ice for 10 min. For each bacterial culture, 10 ml was taken and centrifuged. The supernatants and the bacterial pellets were used for preparation of extracellular and total cellular proteins respectively. The supernatants were filtered with 0.2 μm syringe filter and precipitated with trichloroacetic acid (TCA) at a final concentration of 10%. The precipitates were pelleted by centrifugation, washed twice with acetone, dried and re‐suspended in SDS sampling buffer. For isolation of total cellular proteins, the bacterial pellets were re‐suspended in PBS buffer and the cells were broken by sonification. After centrifugation, the supernatants which contain total cellular proteins were kept for further analysis. The protein samples were denatured by boiling for 5 min and separated by 10% SDS‐PAGE. Western blot analysis was performed following the standard protocol.

### *In situ* detection of Spd in mammalian cell lines

Human lung carcinoma cell line A549 was seeded on the coverslip in six‐well culture plate for 12 h, and the coverslip was washed with PBS buffer. The cells were then fixed by using chill methanol at −20°C for 10 min. After washing with PBS buffer, the cells were incubated with primary antibody Mab 4E4 or control IgG for 1 h prior to washing three times with the same buffer. The labelled second antibody (Alexa fluor 594, invitrogen) was then added at room temperature and incubated for 1 h. After washing, the samples were mounted and observed under a confocal microscope.

### Bacterial inoculation

*Pseudomonas aeruginosa* PAO1 was cultured overnight in LB medium prior to dilution to an OD_600_ of 0.1 in fresh LB medium and re‐incubation for an additional 4 h at 37°C. Cells were harvested by centrifugation and the cell pellet washed twice with sterile PBS prior to re‐suspension in sterile PBS to obtain a final working stock concentration of 3 × 10^7^ colony‐forming units (CFUs) ml^−1^. Fifty microlitres of the bacterial suspension were instilled per mouse (final amount of 1.5 × 10^6 ^CFU, equal to 3× the lethal dose) into randomly sampled Balb/c mice about 8–10 weeks of age. Mouse survival was scored daily for 7 days, and the experiment was repeated at least three times. Every time each group 10 mice were used. The use of live animals and the experimental protocols were approved by the Institutional Animal Care and Use Committee (IACUC) of the Agency for Science, Technology and Research, Singapore.

### Murine infection models

Dose‐dependent survival assays were performed using control IgG (IgG1) and Mab 4E4 dilutions (from 50 μg to 2 mg in a total volume of 100 μl) injected through the tail vein of mice 2 h prior to the instillation of *P. aeruginosa* PAO1 (1.5e10^6^ CFU). The mean survival time was calculated for each treatment group. control IgG (IgG1) and Mab 4E4 (2 mg) was injected respectively through the tail vein of mice 2 h ahead or 2 h after or at the same time with bacterial inoculum. In addition, IgG1 alone (control) and Mab 4E4 (2 mg) was also injected respectively through intraperitoneal (i.p.), intravenous (i.v.) or intrathecal (i.t.) to compare the efficacy of Mab delivery. Balb/c were used for experiments, every time each group the number of mice was 10, totally each experiment were repeated three times. Data are average ± SD and are analysed by using Prism7 GraphPadby.

### Lung histology

Following euthanasia with avertin (500 mg kg^−1^), the thoracic cavity was opened in order to expose the heart and lungs. The trachea was cannulated with PE 90 tubing and suture tightened after the infusion of 0.5 ml of air to expand the lungs. The lungs were then isolated and immersed in 10% formalin for histology. Haematoxylin and eosin (H&E) staining was performed on paraffin‐embedded slides. Neutrophils were counted in triplicate microscopic fields per sample. The lung injury score was determined in a blinded manner.

## Conflict of interest

None declared.

## Supporting information


**Fig. S1.** Schematic diagram of spermidine (Spd) coupling with carrier proteins. A similar reaction was used to prepare OVA–Spm conjugate.
**Fig. S2.** SDS‐PAGE electrophoresis analysis of the purified Mab 4E4. The molecular markers (ladder) are in the left lane.
**Fig. S3.** Monoclonal antibody isotype determination using supernatants of hybridoma.
**Fig. S4.** The chemical structures of polyamines used in this study. The motif recognized by Mab 4E4 is indicated.
**Fig. S5.** Characterization of monoclonal antibody Mab 4E4. (A) Mab 4E4 recognized Spd and Spm, but not OVA. (full film of Fig. 2A).
