# Supplementary material for: Immunological blocking of spermidine‐mediated host–pathogen communication provides effective control against Pseudomonas aeruginosa infection
Source: Microb Biotechnol. 2018 May 14;13(1):87–96. doi: 10.1111/1751-7915.13279 (PMC6922524; doi:10.1111/1751-7915.13279)

**SUPPORTING INFORMATION**

**Fig. S1.** Schematic diagram of spermidine (Spd) coupling with carrier proteins. A similar reaction was used to prepare OVA-Spd conjugate.


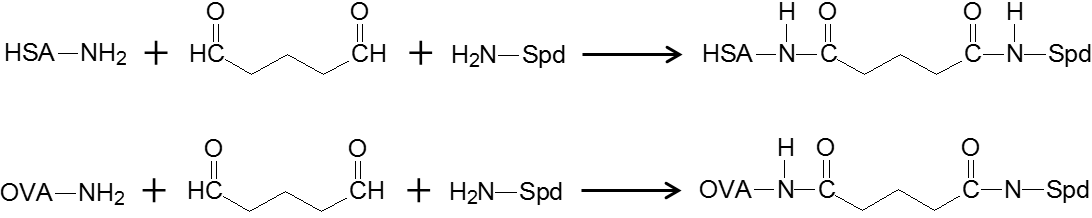


**Fig. S2.** SDS-PAGE electrophoresis analysis of the purified Mab 4E4. The molecular markers (ladder) are in the left lane.


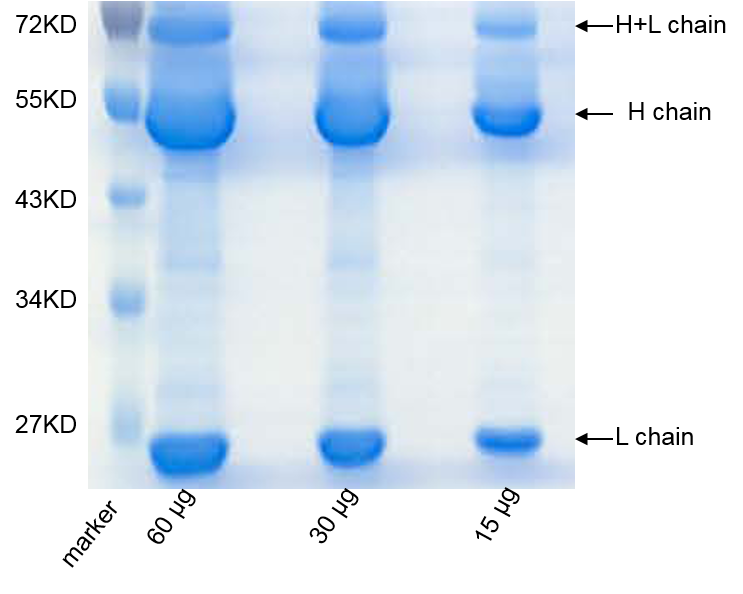


**Fig. S3.** Monoclonal antibody isotype determination using supernatants of hybridoma.


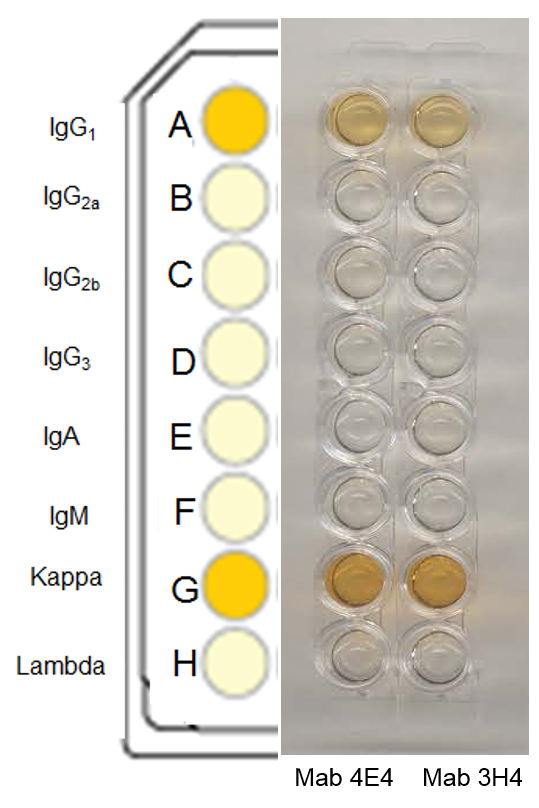


**Fig. S4.** The chemical structures of polyamines used in this study. The motif recognized by Mab 4E4 is indicated.


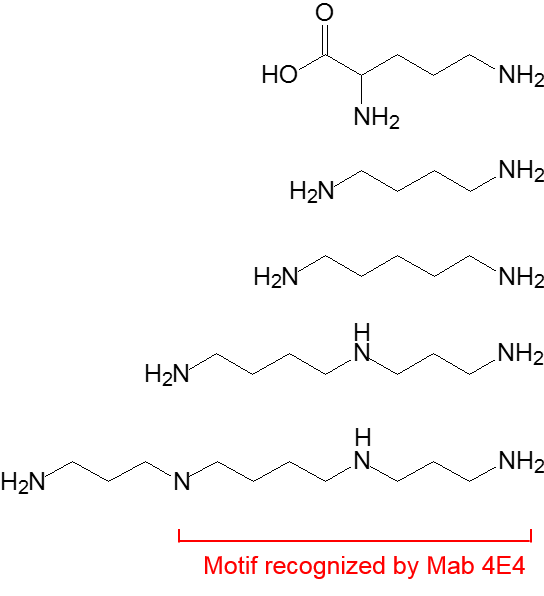


S5. Characterization of monoclonal antibody Mab 4E4. (A) Mab 4E4 recognized Spd and Spm, but not OVA. （full film of fig2A）.

.
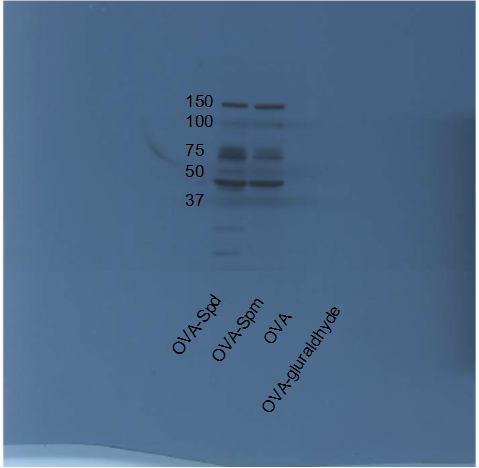

Supplement: Supplementary file 1 — Fig. S1. Schematic diagram of spermidine (Spd) coupling with carrier proteins. A similar reaction was used to prepare OVA–Spm conjugate. Fig. S2. SDS‐PAGE electrophoresis analysis of the purified Mab 4E4. The molecular markers (ladder) are in the left lane. Fig. S3. Monoclonal antibody isotype determination using supernatants of hybridoma. Fig. S4. The chemical structures of polyamines used in this study. The motif recognized by Mab 4E4 is indicated. Fig. S5. Characterization of monoclonal antibody Mab 4E4. (A) Mab 4E4 recognized Spd and Spm, but not OVA. (full film of Fig. 2A). [file MBT2-13-87-s001.docx]
